# Stigma of living as an autism carer: a brief psycho-social support intervention (SOLACE). Study protocol for a randomised controlled feasibility study

**DOI:** 10.1186/s40814-019-0406-9

**Published:** 2019-02-26

**Authors:** Annemarie Lodder, Chris Papadopoulos, Gurch Randhawa

**Affiliations:** Institute of Health Research, University of Bedfordshire, Putteridge Bury, Hitchin Road, Luton, LU2 8LE UK

**Keywords:** Autism, ASD, Family carers, Intervention, Parents, Psychological well-being, Stigma

## Abstract

**Background:**

Stigma is prominent in the lives of autistic individuals and their families and contributes significantly to the challenges faced by families raising an autistic child. Parents and carers can feel blamed for their child’s behaviour, feel socially excluded and isolated and suffer from low self-esteem and poor psychological well-being. This increases the risk of experiencing self-stigma which further exacerbates these and other negative consequences. Therefore, there is a need for interventions that help parents/family carers cope with autism-related stigma as well as prevent the internalisation of stigma.

**Objectives:**

The primary objective of this study is to assess the feasibility and acceptability of a stigma support intervention for parents and carers of autistic children titled ‘Stigma of Living as an Autism Carer (SOLACE)’. The secondary objective is to explore the preliminary impact of the intervention on the mental health of the parents and carers.

**Methods:**

A pilot randomised controlled trial feasibility study will be implemented. A group receiving the SOLACE stigma support intervention (*n* = 12) will be compared against a control group not receiving any additional intervention (*n* = 12). Family carers of autistic children up to the age of 10, who have been recently diagnosed or are currently undergoing diagnosis, will be recruited for the study. Participants will be randomly allocated to the intervention or control group and will take part in eight weekly group-based sessions designed to improve the well-being of the parents primarily through increasing their resilience to stigma. Feasibility will be determined by recruitment and retention rates and a qualitative focus group evaluating the acceptability of the intervention and outcome measures. The primary outcome of interest is psychological well-being, and depending on the normality of distribution, independent samples *T* tests will be used to compare the outcome scores between the two groups and dependent samples *T* tests for differences within the group. Other outcomes of interest are stigma, self-stigma, self-esteem, self-blame, social isolation, self-compassion and perceived responsibility and control.

**Discussion:**

Results from the feasibility randomised controlled trial will be used to refine the study protocol and inform the design of an intervention for future use in a larger, powered trial. SOLACE could potentially improve the psychological well-being of parents/family carers of autistic children through increased resistance to stigma.

**Trial registration:**

ISRCTN Registry number ISRCTN61093625 (October 13, 2017).

**Electronic supplementary material:**

The online version of this article (10.1186/s40814-019-0406-9) contains supplementary material, which is available to authorized users.

## Background

Autism Spectrum Condition (ASC) is a neurodevelopmental condition characterised by diagnostic professionals as impacting upon social interactions, verbal and nonverbal communication, and a narrow spectrum of interests and behaviours [1]. About 1 in 68 children is reportedly on the autism spectrum according to estimates from CDC’s Autism and Developmental Disabilities Monitoring Network [2].

Parents of autistic children report higher parenting stress and lower psychological well-being than the general population [3] and compared to parents of children with other disabilities [4, 5]. The socially challenging behaviours associated with autism contribute to the difficulty of raising an autistic child [6] but so does the public response to this behaviour. Parents in numerous studies report finding the stigma surrounding autism particularly challenging [7–9]. Parents report feeling blamed for their child’s atypical behaviour [10, 11] which subsequently undermines their confidence in their parenting abilities and negatively affects their self-esteem [12, 13]. Negative reactions and attitudes from others also lead families to feel socially isolated [14] and socially excluded [15].

Stigma is prominent in the lives of families with an autistic family member, with up to 95% of families reporting to experience some form of stigma [15–17]. Ali et al. carried out a systematic review of 20 studies to assess the impact of stigma on the well-being of carers and parents of children with intellectual disabilities, including autism [18]. Their results demonstrated that stigma affects the psychological well-being in parents and leads to an increase of parental stress and increased caregiver burden. A recent systematic review carried out by Papadopoulos et al. [19] explored the relationship between autism-related stigma and the mental health of informal caregivers. The review, which assessed nine studies with diverse study designs from across varied socio-cultural settings, consistently found that stigma has a harmful impact on the mental health of caregivers. For example, autism-related stigma was found to be directly related to depression [12, 20], anxiety [21] and psychological distress [22].

A relatively new concept in the autism stigma literature is that of ‘self-stigma’ among parents and family carers. Self-stigma among parents/carers has been defined as the phenomenon by which the public’s negative stereotypes towards both autistic people and, crucially, their family carers, subsequently becomes accepted by parents/carers and incorporated within their own psychological identity [23]. Interviews with parents of autistic children in studies from Cashin [24] and Woodgate, Ateah and Secco [14] have demonstrated how parents of autistic children experience changes to their sense of self and their identity. Self-stigma among parents/carers has also been referred to as ‘affiliate stigma’ [25]. Ntswane and Van Rhyn [26] explored affiliate stigma in a group of South-African mothers of children with intellectual disabilities including autism and found that mothers feel shame, anger, fear, frustration and disappointment. Affiliate stigma has been linked to reduced self-esteem, reduced hope and self-efficacy, reduced quality of life and social avoidance [27, 28].

Parents and carers are recognised as crucial for the well-being of children with disabilities [29, 30]. Given the evidence that connects stigma with the mental health and well-being of parents and carers [19], we theorise that increasing the resilience to stigma will have a positive impact on their well-being and subsequently their caregiving abilities. The development of interventions to reduce self-stigma is still a relatively new area of research. Furthermore, early interventions focused on new carers could be particularly important since they may be more vulnerable to self-blame during the early stages of diagnosis and, as such, to affiliate stigma. New carers are more vulnerable to the misconceptions associated with autism given this may be the first time they have ever encountered autism [19]. Therefore, they may be more prone to misconceptions, myths and negative stereotypes [31].

To our knowledge, there are currently no intervention programs available that help carers of autistic children protect against and cope with the stigma they experience. The aim of the current study is to address this unmet need of carers of autistic children and to contribute to this important gap in the literature. Because there are currently no interventions available, it is unknown what will be effective, acceptable and achievable with regards to the logistics and practicalities of such intervention. It is, therefore, necessary to carry out a feasibility study to assess the feasibility and acceptability of the intervention for a future larger trial. This proposal describes the protocol of a feasibility study designed to evaluate the feasibility and acceptability of a brief psycho-social support intervention, titled ‘Stigma of Living as an Autism Carer (SOLACE)’, for parents/carers of young autistic children. The secondary objective of the study is to evaluate the preliminary results regarding the impact this intervention produced on mental health.

## Methods

### Design

This study is a feasibility study of a parallel randomised controlled trial (RCT) that compares changes in outcomes of interest among parents/carers allocated to the SOLACE experimental group against parents/carers allocated to a control group (no intervention). Standard Protocol Items: Recommendations for Interventional Trials (SPIRIT) checklist was used to structure and develop this protocol.

### Study setting

The study is based in the county of Bedfordshire (UK). Parents and carers will be recruited with help from Autism Bedfordshire, the largest autism charity in the county, and the Luton Borough Council Special Needs team by advertisements in their newsletter, their website and social media channels. The study will be advertised on the University Institute of Health Research’s website and social media channels as well as on the researcher and supervisors’ personal social media accounts. Parents and carers who are interested in taking part or finding out more information are invited to access the URL link to an online information sheet or can contact the research team directly. Digital informed consent will be sought before directing the participants to the baseline assessment.

### Study population and eligibility criteria

This study targets parents and carers of autistic children up to the age of 10 years who have been diagnosed within the past 12 months or are currently undergoing assessment. The participant inclusion criteria are the following: parents or carers aged 18 years or older who care for an autistic child aged up to 10 years and who reside within the county of Bedfordshire. Participants must be able to speak and understand English and are required to have access to the internet or a device able to conduct video-conferencing (e.g. mobile, tablet, PC with a camera and microphone). Individuals who do not fully meet the above criteria are excluded. Our goal is to recruit participants from Bedfordshire; however, if we are unable to recruit a sufficient number of participants from this area, participants from neighbouring counties may also need to be included.

### Sample size

As this is a feasibility study, it is not powered to determine statistical significance. The target recruitment for this feasibility trial is 24 (12 per group). This number is deemed appropriate for the study aims and consistent with recommendations for behavioural group intervention with this population [32].

### Randomisation and blinding

After completing the baseline questionnaire, all participants eligible to take part will be stratified based on gender and frequency of use of support groups (ranging from 0 (never) to 4 (often)) to minimise imbalance between the two groups. The participants will be randomised by an independent researcher via computer generated allocation using JavaScript’s Math functions [33] to either the SOLACE intervention or to the control group. Double-blinding of group allocation is not practically possible since the intervention will be facilitated by the researchers. Figure S1 illustrates the recruitment process and can be found in Additional file 1.

### Intervention: SOLACE

The intervention comprises of eight sessions and include theory, group discussions, short video clips, exercises and examples of parents/carers’ experiences. The intervention will be facilitated by the first author with the support of a voluntary assistant facilitator who will be recruited from the university’s Masters in Public Health course. During the intervention period, a ‘secret’ Facebook group, which only the experimental group participants will have access to, will be used to support the intervention and to boost retention rates. This will be administered and moderated by the lead facilitator. To further boost retention rates, weekly Facebook private messaging reminders will be sent to the experimental group participants.

### Development of the intervention

The intervention is designed and developed based on theoretical evidence, input from key stakeholders and the target population and follows the guidelines from the Medical Research Council for the development and evaluation of complex interventions. [34]. A review from the literature and a systematic review from Papadopoulos et al. [19] identified the psychosocial variables which mediate the relationship between stigma and mental health for family carers of autistic children. Identification of modifiable psycho-social variables has led to the development of a theoretical framework with key variables to be targeted in the intervention. This is illustrated in Additional file 2: Figure S2.

To encourage input from the autism community, an online survey was carried out among the autism community (*n* = 112) about their views and suggestions to make an intervention more successful. Thematic analysis of the responses revealed that respondents suggested that parents’ self-esteem should be boosted, and that they would benefit from ‘ready-made’ phrases or information available to react to instances of stigma from the public, other family members and professionals. Respondents also showed a real desire to connect with other carers and to be able to share experiences and feelings. They explained however that childcare would be a barrier to attendance which is why a blend of online and face to face sessions has been chosen as the optimal mode of delivery.

### Content

The focus of the content will be on autism stigma and how to cope with this. A respectful and supportive environment will be encouraged where parents/carers can share their experiences and support and learn from each other. A description of the content of the sessions is shown in Table 1.

**Table 1:** A brief overview of SOLACE

Number	Topic	Theme	Aim
1	Introduction	Exploration of autism and autism stigma. Autism myths and stereotypes will be challenged through psychoeducation and a group discussion about common stereotypes.	To make introductions and provide an overview of the sessions. To increase knowledge about autism. To reduce feelings of self-blame and increase self-esteem.
2	Coping with autism stigma	Group discussions of experiences of stigma using quotes from other parents. “How would you treat a friend” exercise.	To develop skills how to recognise and cope with stigma and to prevent internalising stigma.
3	Positive meaning of caregiving	Video clips of parents of autistic children showing how having an autistic child has changed them. Group discussion based on that. Free sharing time.	To increase the positive meaning associated with the caregiving role and increase self-compassion.
4	Resilience & self-esteem	Group task to work together to find “key phrases and responses” to stigmatising situations.	To increase resilience and increase self-esteem. To reduce social isolation and increase feeling of belonging.
5	Social support	Stress the importance of social support and discuss together how social support could be utilised and or improved.	To stress the importance of social support to help reduce social isolation.
6	Compassion & acceptance	Discuss the importance of self-care and self-compassion as well as acceptance of the child. Use video clips of how other parents have achieved this.	To increase feelings of self-compassion and acceptance.
7	Coping with autism stigma part 2.	Discuss the automatic thought cycle (self-fulfilling prophecy). Group discussion on how we can break this cycle.	To further develop skills how to recognise and cope with stigma and prevent internalisation of stigma
8	Next steps	Group discussions on how to disclose the diagnosis to others and to provide list of support for future.	To further increase self-esteem and reduce social isolation To conclude the sessions and provide “next steps” for future reference
9	Focus group	Group discussion evaluating SOLACE	To evaluate the acceptability and practicality of SOLACE and the outcome measures.

### Format

Three of the intervention sessions will be face to face (session, 1, 4 and 8) and the remaining sessions will be delivered via videoconference to overcome potential barriers that parents and carers are likely to experience such as time commitment, travel and childcare issues. It has been decided that eight sessions should offer the right balance between time commitment and being beneficial.

### Control group

Participants allocated to the control group will be asked to continue with their normal day to day life as usual. Measures will be taken again post-intervention period (week 8) and 6 weeks post intervention (week 14) to describe potential preliminary differences between the control and intervention group. After the study period, an information pack including useful information and activities that were part of SOLACE will be provided to this group.

### Outcome measures

Continuation criteria will be used to assess if further evaluation of this intervention is justified, through a formal RCT. The criteria for continuation will be based around feasibility and acceptability. A mix of quantitative and qualitative evaluation processes will be used to evaluate the feasibility of the intervention.

The outcome measures to assess feasibility and acceptability are the following:

1. Recruitment rate, including willingness to be randomised.
2. Drop-out rates including reasons will be recorded to help determine the acceptability of the intervention to participants.
3. Missing data will be descriptively analysed in order to evaluate the acceptability of the data collection measures and procedures.
4. Incidence of adverse events will be recorded that may arise during the intervention period.
5. To evaluate the acceptability of SOLACE and the outcome measures, participants from the intervention group will be invited for a qualitative focus group discussion in week 9. The focus group interview will be audio-recorded for analysis. A topic list will be used to evaluate the intervention content, structure, format and mode of delivery. Positive quality feedback of participants regarding their experience of undertaking SOLACE should indicate whether a larger trial may be feasible.

### Primary outcome measure

#### Psychological well-being

Psychological well-being will be measured with the validated and reliable 5-item ‘Mental Health Inventory’(MHI-5) [35] . Participants are asked to rate on a six-point scale how often they felt as described during the past month, e.g. ‘During the past month, how much of the time were you a happy person’. The MHI-5, which is used as the measure of common mental disorder, has excellent validity and reliability in measuring symptoms consistent with depression and anxiety [36] and has a long history of use in large-scale health studies [37], including within the caregiver population [36].

#### Secondary outcome measures

Public stigma will be measured using the Perceived Public Stigma Scale [38]. This scale contains eight items on a six-point Likert scale ranging from 0 (strongly disagree) to 5 (strongly agree) with higher scores indicating higher levels of perceived public stigma. A sample statement is ‘Most people think less of an autistic person than they do of other people’. This scale has been used with carers and parents of autistic children and shown good reliability (*α* = 0.83) [38].

#### Courtesy stigma

The Perceived Courtesy Stigma Scale (PCSS) [38] contains seven items adapted from the Devaluation of Consumer Families Scale [39]. Caregivers rate each item on a four-point Likert scale ranging from 0 (strongly disagree) to 3 (strongly agree) with higher scores suggesting higher levels of perceived courtesy stigma. The PCSS has been used with parents of autistic children and its validity was demonstrated by its significant correlations with theoretically related constructs [38] and a good reliability score of *α* = 0.89 [38]. Items include statements like ‘most people blame parents for their child being autistic’.

#### Affiliate stigma

Affiliate stigma is measured with the Affiliate Stigma Scale [25]. This 22-item scale has been adapted by the study team to be suitable for UK caregivers. Three items with the lowest factor loadings have been dropped in order to decrease the number of negatively worded items based on work by Werner and Shulman [28]. Each item is rated on a 4-point Likert scale (1, strongly disagree to 4 strongly agree).

#### Self-esteem

Rosenberg’s 10-item Self-Esteem Scale [40] is used to measure self-esteem. An overall index can be calculated by averaging the score on the 10 items scored on a 4-point Likert scale. The Rosenberg Self-Esteem Scale is a widely used measure of self-esteem which has been shown to have good internal consistency in various populations.

#### Self- compassion

Self-compassion is measured with the Self-Compassion Scale-Short Form [41], a modified version of the Self-Compassion Scale [42]. It measures six components of self-compassion: self-kindness, self-judgement, common humanity, isolation, mindfulness, and over-identification. The 12 items are rated on a 5-point response scale ranging from 1 (almost never) to 5 (almost always). The SCS-SF has demonstrated good internal consistency (*α* ≥ 0.86) and a near-perfect correlation with the long-form self-compassion scale (SCS) (*r* ≥ 0.97) [41].

#### Positive meaning of caregiving

Based on Werner and Shulman’s [28] study, positive meaning in caregiving will be measured with 11 items taken from two sources: from parents’ qualitative responses to the question ‘How has caregiving for your child affected your life?’ in a study by Meyers et al. [43] and from the Perceived Benefits scale constructed by Green [44] containing 5 items. A sample item is ‘Being a parent/carer to an autistic child with a taught me kindness, patience and happiness’.

#### Self-blame/responsibility

The Self-blame and Responsibility Scale has been translated from Chinese by Mak and Kwok [27] and has been slightly adapted to make it more suitable for the UK population and the wording has been changed to ‘autism-first language’ as is generally preferred by the autism community.

#### Social Support

To measure perceived social support, the Medical Outcomes Study: Social Support Survey [45] will be used. This scale measures four components of perceived availability of social support, including (1) emotional support/informational support, (2) tangible support (including material support), (3) positive social interaction (does person have friends that are available to have fun) and (4) affectionate support (including loving and nurturing relationships). It has shown good reliability (*α* = .85) and validity on total scale (*α* = .88) as well as subscales.

#### (Subjective) social isolation

The short-form UCLA Loneliness Scale [46] will be used to measure social isolation. The UCLA Loneliness Scale has been found to be a highly reliable measure—both in terms of internal consistency and test–retest reliability over a one-year period—and to have convergent and construct validity [46].

#### Other baseline measures

Information on general demographic variables such as age, gender, ethnicity, marital status, income, religious affirmation, educational level, child’s primary diagnosis, number of children, age of child, time since diagnosis and current use of support groups.

### Statistical analysis

Statistical analysis (using IBM SPSS v23 software) of the quantitative outcome measurements will be conducted following tool analytical guidance. Within group comparisons of the outcomes, measures will be made as well as between groups comparisons to examine preliminary effectiveness.

Kolmogorov–Smirnov tests will be initially conducted to establish the normality of the data. Depending on the normality of distribution, independent samples *T* tests, Mann–Whitney *U* tests and/or chi-square tests will be used to compare the outcome measures between the two groups. To assess potential within-group differences, dependent *T* tests (for normal distribution) or Wilcoxon signed-rank tests (for non-normal distribution) will be used. Individual and group-level descriptive analyses of both the frequency and/or central distribution (medians, means, standard deviation, range) of independent and dependent variables will also be conducted. Standard effect sizes of the primary outcome measure will be calculated (Cohen’s *α*) with confidence intervals to provide an initial estimate for sample size calculation for a future main RCT.

Furthermore, quantitative data will be descriptively analysed to assess rates and patterns of recruitment, follow-up and retention rates, and willingness of randomisation. The distribution of missing data within each measure will help to establish the acceptability of measures. Missing data will also be described via frequencies and cross tabulations against background characteristics to further assess the acceptability of the measurement tools.

Qualitative data will be thematically analysed using Nvivo (v11). This will involve in-depth familiarisation with the focus group data, systematic identification of codes to form a coding frame, indexing the data according to the coding frame to identify common themes and interpreting the findings in the context of other research [47].

The Consolidated Standard of Reporting Trials (CONSORT) extension to pilot trials will be followed for reporting the trial.

### Data monitoring and management

All study data will be captured electronically in a secured online survey platform (www.qualtrics.com). The data will then be uploaded into the IBM SPSS database. Qualitative data will be collected using audio recordings, written notes and memos. The program Nvivo will be used to analyse the qualitative data. Recordings of the online intervention will be securely stored on the researcher’s password protected laptop and only used to assess fidelity to the intervention manual during meetings with the research team. All data will be anonymised and electronically stored at the University of Bedfordshire, separate from identifying information. Access to data will be password-protected. Only the research team will have access to the final dataset. All collected data will be used only for the purposes of this research.

### Ethical considerations

This study is being conducted in accordance with the ethical guidelines of the Institute of Health Research to ensure trial integrity as well as participants’ safety and well-being. Ethical approval was granted on January 1, 2018 (ref: IHREC812), from the University of Bedfordshire, Institute of Health Research’s ethics committee.

Respondents who are interested in taking part will receive detailed information about the study and have the opportunity to ask questions. Digital informed consent will be obtained from all participants before the start of the study. Participants are allowed to withdraw from the study at any time and it will be made clear that their anonymity will be respected at all times in the dissemination of results.

### Harms

The intervention is considered safe and there is no evidence to suggest that the planned intervention will be reasonably likely to cause any harm to participants. It is anticipated that the benefits of the study will be greater than any possible risk. Participants will be told that in the event that they do feel distress, they can withdraw from the study at any time. In such cases, we will offer participants the opportunity to debrief with the facilitator, as well as a list of caregiver mental health resources (including services across the county and relevant online support groups). Likewise, it is unlikely that participants will experience any harm during the focus group interview given that the focus will primarily regard the feasibility, practicality and acceptability of the intervention. It is ensured that all questions and probes used are phrased sensitively. Any adverse events that do occur will be recorded and reported when necessary. A protocol and record log for adverse events will be utilised.

## Discussion

The study described here aims to determine the feasibility and preliminary impact of an 8-week psycho-social stigma support intervention, ‘SOLACE’, with a group of parents and carers of newly diagnosed autistic children. To our knowledge, this study will be the first RCT to evaluate the feasibility and preliminary effects of a stigma support intervention for parents/carers of autistic children. As previously highlighted, parents/carers of autistic children are particularly vulnerable to poor mental health and well-being, and the stigma of autism likely significantly contributes to this. Therefore, an intervention that is evidenced to improve psychological well-being in part through increasing resistance to stigma will be of substantial benefit to families and, ultimately, their children. This study represents the first step towards achieving this goal. The learning generated from this study will enable the preparation for a future larger trial that can more confidently assess SOLACE’s effectiveness in improving psychological well-being.

The strength of the study design is the inclusion of a qualitative analysis to assess the feasibility of the intervention. Further, this feasibility study will provide a reliable indication of the sample size needed for an RCT testing the effectiveness of the intervention. It will also address a large gap in the autism-stigma literature since not much is known yet about the path between stigma and psychological well-being. However, as usual with feasibility studies, only preliminary conclusions on the effectiveness of the intervention can be drawn, owing to the small sample size.

The carer population is known to experience many barriers to attendance. SOLACE aims to overcome this barrier by delivering a combination of face to face and online sessions. This should also mean it is relatively easy to implement and to replicate in the future. If SOLACE is feasible and eventually proven to be effective, then health and social care services can commission and include the intervention as part of their service provision. The evidence may also be used to help shape future local and national (and potentially international) autism policy so that the importance of stigma in relation to caregivers’ psychological well-being and their caregiving role is emphasised.

## Additional files

Additional file 1: Figure S1**.** Recruitment and retention flowchart. (JPG 63 kb)

Additional file 2: Theoretical framework of the stigma-mental health relationship. (PDF 92 kb)

## Abbreviations

ASC: Autism Spectrum Conditions
MHI-5: 5-item Mental Health Inventory
PCSS: perceived consumer stigma scale
RCT: Randomised controlled trial
SCS: Self-compassion scale
SCS-SF: Self-compassion scale – short form
SOLACE: Stigma of Living as an Autism Carer
UK: United Kingdom
