# Supplementary figures and images for: Stigma of living as an autism carer: a brief psycho-social support intervention (SOLACE). Study protocol for a randomised controlled feasibility study
Source: Pilot Feasibility Stud. 2019 Feb 26;5:34. doi: 10.1186/s40814-019-0406-9 (PMC6390626; doi:10.1186/s40814-019-0406-9)

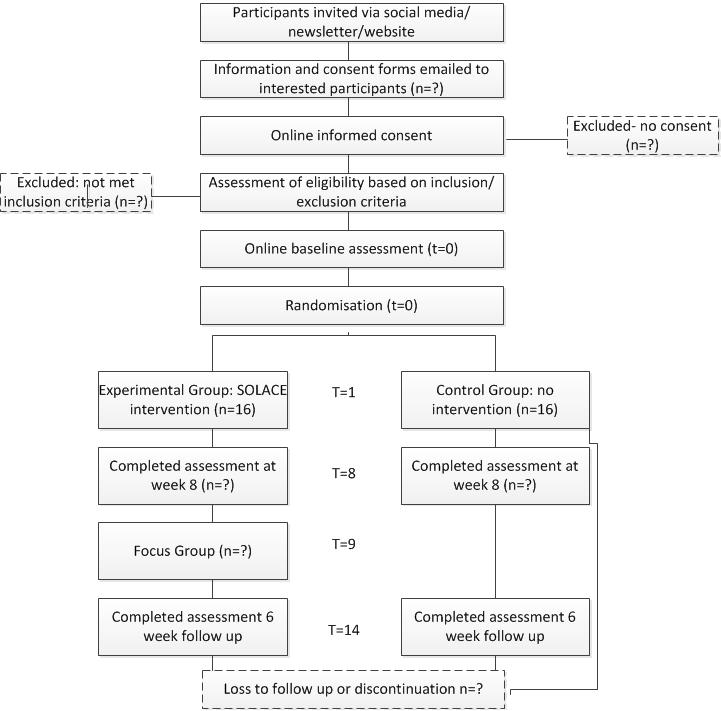

Supplement: Supplementary file 1 — Figure S1. Recruitment and retention flowchart. (JPG 63 kb) [file 40814_2019_406_MOESM1_ESM.jpg]

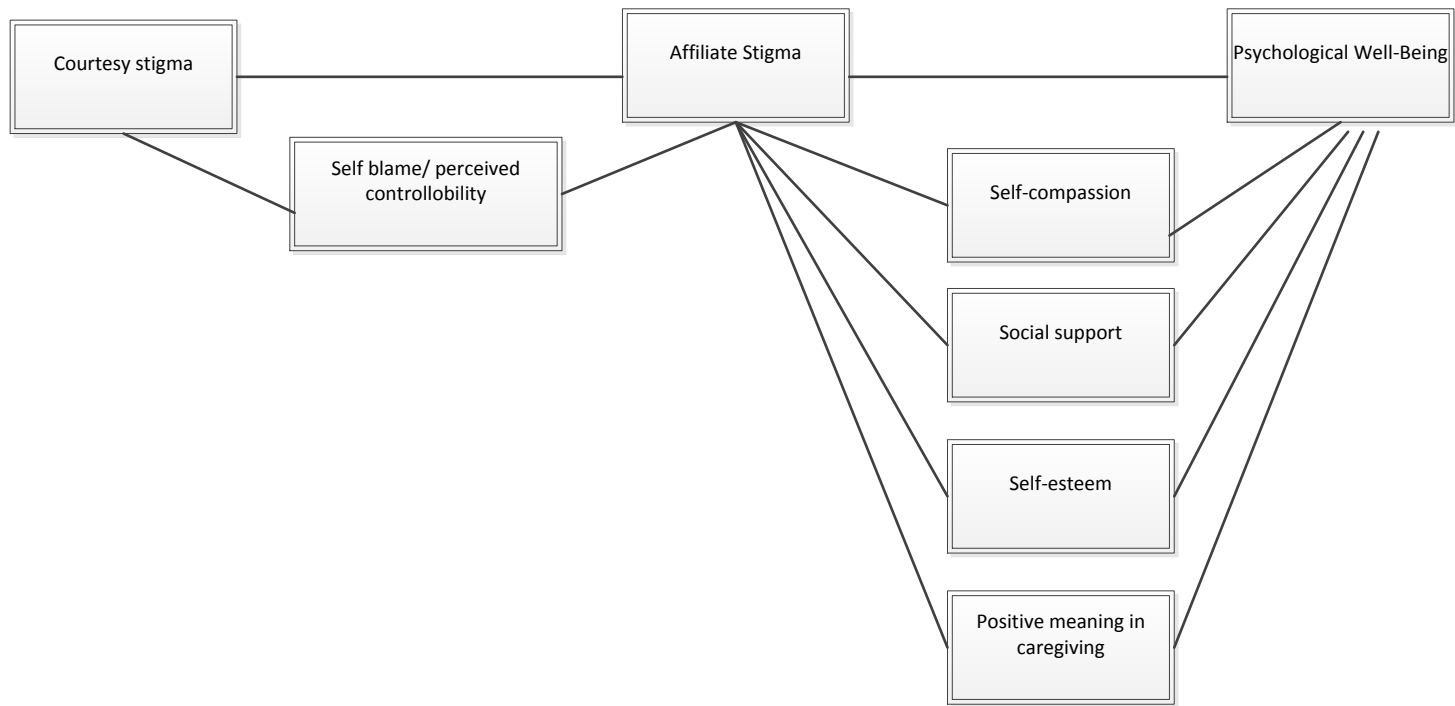

Supplement: Supplementary file 2 — Theoretical framework of the stigma-mental health relationship. (PDF 92 kb) [file 40814_2019_406_MOESM2_ESM.pdf]
